# Pharmacokinetic/pharmacodynamic target attainment of eravacycline in a liver transplant recipient with refractory VRE *faecium* infection

**DOI:** 10.1128/aac.00116-26

**Published:** 2026-05-28

**Authors:** Guanghui Chen, Yinghuan Dai, Qiang Qu, Yiwen Xiao, Feng Wang, Jian Qu

**Affiliations:** 1 Department of Pharmacy, the Second Xiangya Hospital, Central South University 618805 , Changsha, China; 2 Department of Clinical Pharmacy, the Central Hospital of Xiangtan (The Affiliated Hospital of Hunan University) 117752, Xiangtan, China; 3 Department of Pathology, the Second Xiangya Hospital, Central South University 618102 , Changsha, China; 4 Department of Pharmacy, Xiangya Hospital, Central South University 618805 , Changsha, China; 5 Hunan Key Laboratory of the Research and Development of Novel Pharmaceutical Preparations, Changsha Medical University 216793, Changsha, China; Houston Methodist Hospital and Weill Cornell Medical College, Houston, Texas, USA

**Keywords:** liver transplantation, vancomycin-resistant *Enterococcus faecium*, eravacycline, therapeutic drug monitoring, pharmacokinetics/pharmacodynamics

## LETTER

Bacterial infections significantly impact outcomes following liver transplantation (LT) (1). Vancomycin-resistant *Enterococcus faecium* (VREfm) has become a common and challenging pathogen in this population due to multidrug resistance and limited treatment options (2, 3). Eravacycline has demonstrated clinical efficacy in bloodstream infections (4) and potent *in vitro* activity against VREfm (5–8). However, clinical pharmacokinetic/pharmacodynamic (PK/PD) data supporting its use in VREfm infections remain limited.

We report the case of a 32-year-old repeat LT recipient with refractory VREfm infections of the abdomen and pleura. Initial therapy with linezolid (600 mg q12h) was discontinued after 10 days due to thrombocytopenia. Daptomycin (10 mg/kg daily) was subsequently administered as monotherapy, but the patient remained febrile with persistently positive cultures. A multidisciplinary salvage strategy was then initiated with eravacycline (1 mg/kg q12h) in combination with daptomycin. After clinical stabilization, daptomycin was stopped, and eravacycline was continued as monotherapy. This approach resulted in microbiological cure, which was confirmed by negative follow-up bile cultures and resolution of radiographic abnormalities, leading to the patient’s successful discharge. At the time of therapeutic drug monitoring (day 30), the patient’s serum albumin was 2.9 g/dL (normal range 3.5–5.0); total bilirubin was 1.8 mg/dL; direct bilirubin was 1.2 mg/dL, and the Child-Pugh score was 7 (class B). No eravacycline-related adverse events were observed throughout the 42 day course.

Eravacycline concentrations in all matrices were quantified using a validated two-dimensional high-performance liquid chromatography method following protein precipitation. The standard curve was linear, over the range of 280–10,873 ng/mL. The area under the concentration-time curve was calculated using the linear trapezoidal rule. The unbound fraction (*f*) was set at 0.286 based on prior reports (9), consistent with the patient’s hypoalbuminemia.

All 17 serial VREfm isolates were susceptible to eravacycline (MIC range 0.032–0.125 mg/L) (Table 1). On day 30 of eravacycline therapy, therapeutic drug monitoring yielded the following concentrations: plasma trough (*C*_min_), 0.37 mg/L, peak (*C*_max_), 2.23 mg/L; bile, 83.17 mg/L; peritoneal fluid (PF), 1.33 mg/L; and pleural fluid, 0.76 mg/L (Table 2). The calculated free-drug area under the concentration-time curve over 12 hours (fAUC_0–12_) was 2.56 mg·h/L (unbound fraction *f* = 0.286). Resulting plasma fAUC_0–24_/MIC ratio of 40.96, and tissue-to-plasma trough concentration ratios in PF (3.59), pleural fluid (2.05), and bile (224.78) were substantially elevated. The concentration-to-MIC ratios were 665× in bile, 10.6× in PF, and 6.1× in pleural fluid.

**TABLE 1:** Antibiotic susceptibility profiles of 17 VREfm isolates obtained during treatment^*a*^

Day	Isolate no.	Specimen type	VAN MIC	TEI MIC	DAP MIC	LZD DD (mm)^*b*^	LZD MIC	TET MIC	MIN MIC	ERV MIC^*c*^
Day 9	1	PF	>32	32	2	27		16	8	0.032
Day 11	2	PF	>32	32	2	27		16	8	0.032
Day 13	3	PF	>32	32	2	27		16	8	0.032
Day 15	4	PF	>32	32	4	30		16	8	0.032
Day 16	5	Pleural fluid	>32	32	4	30		16	8	0.032
Day 18	6	Pleural fluid	>32	32	4	30		16	8	0.032
Day 20	7	Pleural fluid	>32	32	4		2	16	8	0.032
Day 20	8	Plasma	>32	32	4	39		16	4	0.125
Day 23	9	Pleural fluid	>32	32	4	30		16	8	0.032
Day 26	10	PF	>32	32	4	30		16	8	0.032
Day 31	11	PF	>32	32	4	30		16	8	0.032
Day 36	12	PF	>32	32	4	30		16	8	0.032
Day 42	13	PF	>32	32	4	31		16	4	0.032
Day 57	14	PF	>32	32	4	31		16	4	0.032
Day 64	15	PF	>32	32	4	31		16	4	0.032
Day 69	16	PF	>32	32	4		4	16	8	0.032
Day 72	17	PF	>32	32	4		2	16	16	0.032

^*a*^ DAP, daptomycin; ERV, eravacycline; LZD, linezolid; MIC, minimum inhibitory concentration (mg/L); MIN, minocycline; PF, peritoneal fluid; TEI, teicoplanin; TET, tetracycline; VAN, vancomycin.

^*b*^ Disk diffusion zone diameter (mm), susceptibility defined as ≥23 mm per CLSI.

^*c*^ Interpretive criteria per EUCAST (susceptible ≤0.125 mg/L).

**TABLE 2:** Therapeutic drug monitoring of eravacycline and key PK/PD parameters^*a*^

Samples	Concn (mg/L）	Tissue-to-plasma ratio	fAUC_0–24_/MIC
Plasma *C*_min_	0.37		40.96
Plasma *C*_max_	2.23		
Plasma *C*_2h_	0.77		
Bile	83.17	225	
Pleural effusion	0.76	2.1	
Peritoneal fluid	1.33	3.6	

^*a*^ The MIC of the VREfm isolate in plasma was 0.125 mg/L. fAUC_0–24 _was calculated from plasma data.

Eravacycline demonstrated potent *in vitro* activity against all serial VREfm isolates in this case, with MICs consistently ≤0.125 mg/L, consistent with published data showing low MICs for eravacycline against VREfm. There is a lack of *in vivo* data of eravacycline against VREfm. Animal infection models have confirmed the *in vivo* efficacy of eravacycline against methicillin-resistant *Staphylococcus aureus* (MRSA) (10, 11). Despite limited data, clinical efficacy of eravacycline against VRE has been documented, with studies reporting success rates between 50% and 58.3% (12, 13).

The fAUC/MIC ratio is the key PK/PD index for eravacycline (14). Although established PK/PD targets primarily concern gram-negative bacteria and MRSA (15–18), in our patient, eravacycline concentrations in bile (~333 × MIC_90_), PF (~5 × MIC_90_), and pleural fluid (~3 × MIC_90_) substantially exceeded the MIC_90_ for common pathogens (0.25 mg/L). Furthermore, corresponding tissue-to-*C*_min_ ratios reached approximately 224.78 in bile, 3.59 in PF, and 2.05 in pleural fluid, directly quantifying tissue penetration. The resultant high fAUC/MIC ratios at these sites not only surpassed MRSA efficacy targets but also exceeded the *Escherichia coli* PK/PD targets (static: 27.97, 1-log kill: 32.60), which was instrumental in achieving cure.

In conclusion, this case demonstrates that PK/PD-guided eravacycline therapy, characterized by excellent tissue penetration and target attainment, represents a successful salvage option for refractory VREfm infections in transplant recipients. Further studies are warranted to validate its efficacy and to optimize dosing strategies in this population.
